# Risk factors for mortality in patients with sepsis on extracorporeal membrane oxygenation and/or continuous renal replacement therapy: a retrospective cohort study based on MIMIC-IV database

**DOI:** 10.1080/0886022X.2024.2436106

**Published:** 2024-12-04

**Authors:** Tongxin Chu, Jinyu Pan, Qingyang Song, Qiushi Ren, Quan Liu, Huayang Li, Liqun Shang, Gang Li, Jian Hou, Suiqing Huang, Zhongkai Wu

**Affiliations:** a Department of Cardiac Surgery, First Affiliated Hospital of Sun Yat-sen University, Guangzhou, China; b Department of Cardiology, The Affiliated Panyu Central Hospital of Guangzhou Medical University, Guangzhou, China

**Keywords:** Extracorporeal membrane oxygenation, continuous renal replacement therapy, sepsis, risk factors, mortality

## Abstract

**Objective:**

This study aimed to identify risk factors for mortality in septic patients undergoing extracorporeal membrane oxygenation (ECMO) and/or continuous renal replacement therapy (CRRT).

**Methods:**

Data from the MIMIC-IV database were retrospectively reviewed for 24,502 septic patients treated with ECMO or CRRT between 2008 and 2019. After applying inclusion and exclusion criteria, 70 patients receiving ECMO, 513 receiving CRRT, and 22 receiving both were included in the final analysis. Univariate and multivariate stepwise Cox regression analyses were performed to identify independent risk factors for mortality. Model performance was assessed using receiver operating characteristic (ROC) curve analysis. We also provided model-agnostic explanations for each Cox regression model.

**Results:**

For septic patients on ECMO, prothrombin time (per 1-s increase, HR 1.037, 95% CI 1.007–1.068, *p* = .015) was the key independent risk factor. For septic patients undergoing CRRT, SOFA score (per one-point increase, HR 1.100, 95% CI 1.055–1.147, *p* < .001) was the most significant factor. For septic patients requiring both ECMO and CRRT, prior history of hypertension (HR 4.342, 95% CI 1.332–14.153, *p* = .015) was the sole independent risk factor. ROC analysis showed satisfactory model performance (AUC > 0.75).

**Conclusion:**

For septic patients requiring ECMO, prothrombin time was the key independent risk factor. For those needing CRRT, SOFA score was the most significant independent risk factor. Prior history of hypertension was the primary independent risk factor for septic patients needing both CRRT and ECMO.

## Introduction

Sepsis is a clinical syndrome characterized by a dysregulated host response to infection, leading to multiple organ dysfunction and death [1]. This study aimed to identify risk factors for the mortality of patients with sepsis undergoing extracorporeal membrane oxygenation (ECMO) and/or continuous renal replacement therapy (CRRT), which is significant for guiding clinical treatment and improving the prognosis of septic patients.

The feasibility of ECMO for adult patients with sepsis-induced respiratory and circulatory failure remains debatable [2]. CRRT is used for extracorporeal blood purification in cases of refractory acute kidney injury (AKI) [3]. Additionally, ECMO’s hemodynamic effects and related factors increase the risk of AKI. In contrast, CRRT’s gradual solute clearance minimally impacts critically ill patients’ hemodynamics, making it more suitable for ECMO-treated patients than traditional hemodialysis. Notably, patients receiving both therapies tend to have higher mortality [4].

Although previous studies have primarily focused on the risk factors for patients with sepsis, there is a scarcity of research on the combined use of ECMO and CRRT. Our previous studies have reported risk factors for mortality in surgical patients on combined CRRT and ECMO [5]. Other studies on combined ECMO and CRRT have mainly focused on coronavirus disease 2019 [6,7]. However, the risk factors for mortality in patients with sepsis treated with ECMO and/or CRRT remain unclear. Hence, there is an evident need for early recognition of modifiable risk factors and effective management strategies to mitigate the risk of death in this population.

Therefore, in this study, we retrospectively analyzed the patients with sepsis undergoing ECMO and/or CRRT from Medical Information Mart for Intensive Care IV (MIMIC-IV) database. We aimed to construct multivariate Cox regression models and investigate the independent risk factors for mortality in patients undergoing ECMO and/or CRRT, which can help us find breakthroughs for high mortality.

## Method

### Data source and study design

The study was performed in accordance with the STROBE statement [8]. Data were extracted from the MIMIC-IV database, an updated MIMIC-III approved by an institutional review board [9]. From 2008 to 2019, 382,278 patients admitted to intensive care units (ICUs) at Beth Israel Deaconess Medical Center (BIDMC) were included in the MIMIC-IV which contains demographics, laboratory indicators, vital signs, and medications. For permission to access the database, the author (Qingyang Song) has accomplished a recognized course in the Protecting Human Research Participants (certification number: 60220134). The individual information of the patients included in this database was anonymous, and the need for ethical review and informed consent was waived.

### Study participants

According to the criteria outlined in ‘Sepsis 3.0’, sepsis is defined as a two-point or more increase in the SOFA score, in conjunction with diagnosed or suspected infections [10]. The target population included adults diagnosed with sepsis undergoing ECMO or CRRT. The exclusion criteria were as follows: (1) age < 18 years, (2) missing data, and (3) pregnancy. Finally, 70 patients treated with ECMO and 513 patients treated with CRRT were included.

### Study outcome

In this study, the follow-up time to determine the end point of death was during the patient’s hospitalization. The primary endpoint was the 28-day mortality after ICU admission.

### Variables extraction

For patients with multiple ICU admissions, only data from the first ICU admission were collected. Patient data within the first 24 h after admission were extracted from MIMIC-IV using Structured Query Language (SQL) and were collected as follows: (1) age, sex, acute physiology score III (APSIII), oxford acute severity of illness score (OASIS), logistic organ dysfunction system (LODS), sequential organ failure assessment (SOFA), Charlson comorbidity score (2) Comorbidities: heart failure, prior history of hypertension, atrial fibrillation, renal insufficiency, chronic obstructive pulmonary disease (COPD), coronary artery disease (CAD), stroke, malignancy; (3) Vital signs: mean arterial pressure (MAP), heart rate (HR), oxygen saturation (SpO2); (4) Laboratory parameters: lactate, creatinine, blood urea nitrogen (BUN), white blood cell (WBC), red blood cell (RBC), red cell distribution width (RDW), hemoglobin, platelet, international normalized ratio (INR), prothrombin time (PT), activated partial thromboplastin time (APTT), aspartate aminotransferase (AST), total bilirubin (TBIL), blood glucose; (5) Treatment: valve surgery, bypass surgery, ECMO, CRRT.

### Statistical analysis

Continuous variables and categorical variables were reported as the mean ± standard deviation (SD), the median (interquartile range), or as frequencies, as appropriate. The Student’ *t*-test or a non-parametric test was used for the analysis of continuous variables. Categorical variables were analyzed using Fisher’s exact test.

Univariate Cox regression analysis was used to determine the variables that may be related to 28-day mortality, including all variables with *p* value < .1. Then, we used the step function (both method, step-by-step selection) from ‘stepcox’ R packages to screen variables to eliminate the interference of confounding factors [11]. The best multivariate Cox regression model was constructed based on minimum Akechi Information Criteria (AIC). This model was used to analyze the risk factors for 28-day mortality, and the results were presented as hazard ratio (HR) with 95% confidence intervals (CIs). We also provide model-agnostic explanations for each Cox regression model to show the transparency of the model. The receiver operating characteristic (ROC) curve analysis was used to assess the predictive ability. The model was considered fair if the AUROC was 0.7–0.8, and good if the AUROC was 0.8–0.9 [12]. Kaplan–Meier curves were plotted with the duration from the enrollment to the last follow-up or death and compared with the log-rank test.

All tests were two-tailed, and *p* < .05 was considered statistically significant. Statistical analyses were conducted using R (version 4.1.2) with additional R packages including ‘Tableone’, ‘stepcox’, ‘survex’, and ‘pROC’.

## Result

### Patient characteristics

**Figure 1.**
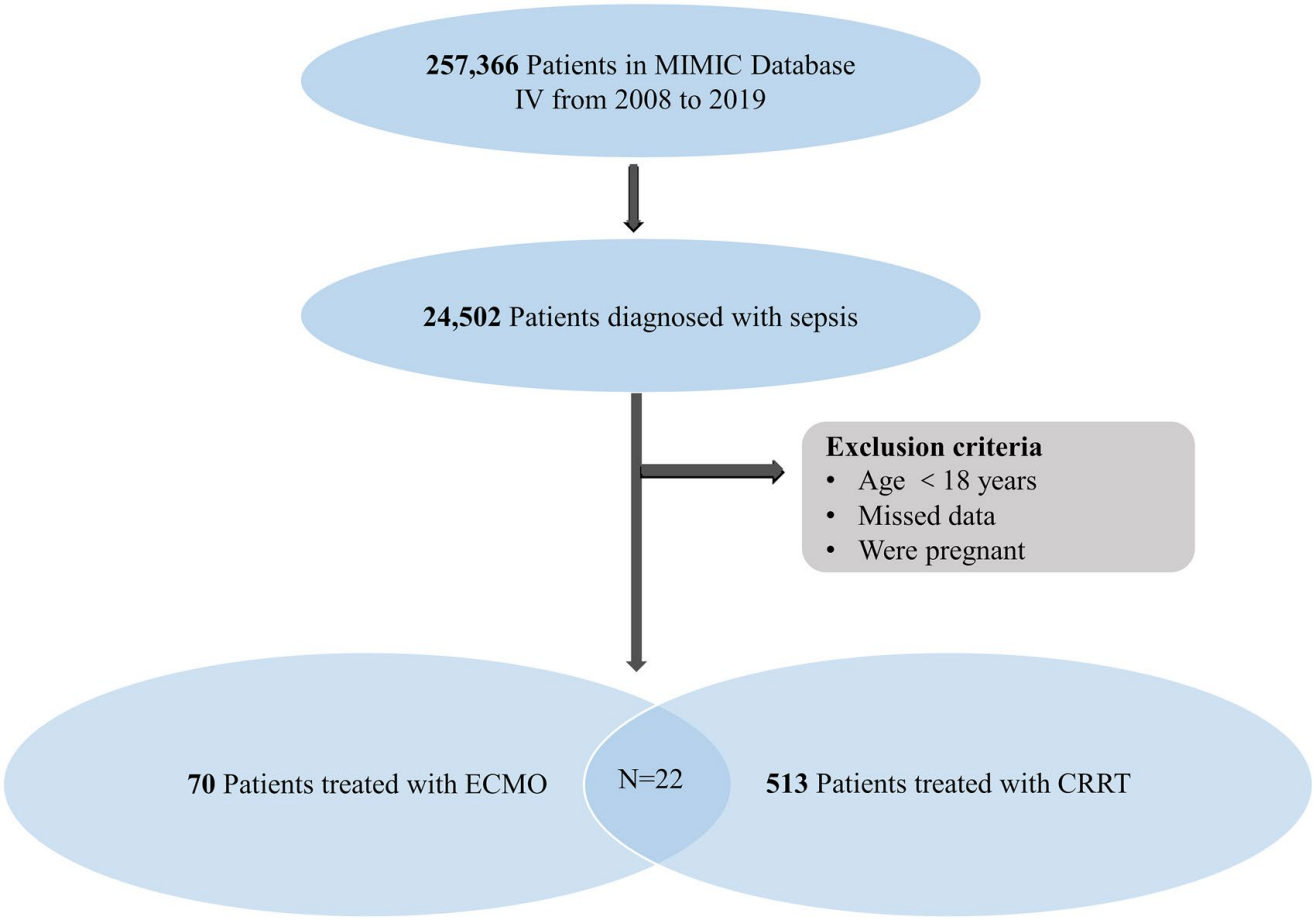
Flow chart for study inclusion/exclusion of patients. ECMO: extracorporeal membrane oxygenation; CRRT: continuous renal replacement therapy.

From 2008 to 2019, a preliminary screening of the MIMIC-IV database identified 23,901 patients with sepsis. Among them, only 70 patients were treated with ECMO implantations and 513 patients were treated with CRRT (Figure 1). The 28-day mortality for patients with sepsis undergoing ECMO support was 60%, while those receiving CRRT treatment had a 55% mortality. For patients with receiving both ECMO and CRRT, the 28-day mortality increased to 73%.

The baseline characteristics of patients with sepsis treated with ECMO were presented in Table 1. The average age of the patients was 52.57 ± 15.72 years, with men accounting for 68.60%. CAD occurred in 27 (38.60%) patients. Additionally, 37 (52.90%) patients had prior history of hypertension, and 15 (21.40%) had renal insufficiency. Compared to those who died, patients who survived had significantly lower Charlson comorbidity score, INR, and PT (*p* < .05). It was also observed that the difference in CRRT treatment between the survival and death patient groups was not statistically significant.

**Table 1.** Baseline clinical data of the included patients with sepsis receiving ECMO support.

Variables	Overall (*n* = 70)	Survival (*n* = 28)	Death (*n* = 42)	*p* Value
Age (years)	52.57 (15.72)	48.32 (16.34)	55.40 (14.83)	.064
Sex, male	48 (68.60)	21 (75.00)	27 (64.30)	.494
APSIII	82.23 (27.30)	80.11 (31.60)	83.64 (24.31)	.599
OASIS	40.91 (8.51)	38.36 (9.03)	42.62 (7.80)	**.039** ^a^
LODS	9.90 (2.87)	9.54 (3.07)	10.14 (2.75)	.391
SOFA	11.81 (3.06)	11.39 (2.82)	12.10 (3.21)	.350
Charlson comorbidity score	4.00 (2.00,6.00)	3.00 (1.00,5.00)	5.00 (3.00,6.00)	**.017** ^a^
Comorbidities, n (%)				
Heart failure	45 (64.30)	17 (60.70)	28 (66.70)	.799
Prior history of hypertension	37 (52.90)	13 (46.40)	24 (57.10)	.525
Atrial fibrillation	7 (10.00)	5 (17.90)	2 (4.80)	.167
Renal insufficiency	15 (21.40)	3 (10.70)	12 (28.60)	.137
COPD	4 (5.70)	0 (0.00)	4 (9.50)	.248
CAD	27 (38.60)	6 (21.40)	21 (50.00)	**.031** ^a^
Stroke	11 (15.70)	2 (7.10)	9 (21.40)	.203
Malignancy	4 (5.70)	3 (10.70)	1 (2.40)	.344
Vital signs				
MAP (mmHg)	73.50 (61.00,83.75)	77.50 (61.00,88.25)	72.50 (60.25,81.25)	.432
HR (bpm)	94.79 (23.02)	96.36 (24.62)	93.74 (22.14)	.644
SpO2 (%)	96.00 (89.50, 100.00)	96.50 (87.00,99.00)	96.00 (92.25, 100.00)	.347
Laboratory tests				
Lactate (mmol/L)	4.75 (2.52,9.10)	3.80 (2.35,6.02)	6.20 (3.00,9.62)	.102
Creatinine (mg/dL)	1.70 (1.30,2.28)	1.60 (1.25,1.92)	1.95 (1.30,2.45)	.242
BUN (mg/dL)	23.50 (17.25,36.50)	22.50 (17.00,29.75)	26.00 (19.00,42.50)	.235
WBC (×10^9^/L)	15.41 (8.69)	14.88 (9.21)	15.76 (8.41)	.682
RBC (×10^12^/L)	3.52 (2.87,4.26)	4.08 (3.52,4.45)	3.17 (2.64,3.92)	**.007** ^a^
RDW (%)	14.28 (13.33,15.47)	13.60 (13.28,14.72)	14.70 (13.67,15.67)	**.023** ^a^
Hemoglobin (g/dL)	10.98 (2.89)	11.87 (2.97)	10.38 (2.71)	**.034** ^a^
Platelet (×10^9^/L)	169.71 (88.37)	184.18 (83.68)	160.07 (91.06)	.267
INR	1.80 (1.40,2.68)	1.60 (1.30,2.05)	1.95 (1.60,2.85)	**.024** ^a^
PT (s)	19.50 (15.45,28.93)	17.45 (14.55,22.18)	21.15 (17.10,30.98)	**.027** ^a^
APTT (s)	77.50 (43.68, 150.00)	76.35 (36.78, 147.38)	81.95 (55.95, 150.00)	.364
AST (U/L)	184.00 (65.75, 695.50)	217.00 (47.25, 525.25)	184.00 (92.75, 778.25)	.657
TBIL (mg/dL)	0.90 (0.50,2.08)	0.85 (0.50,1.68)	0.95 (0.60,2.45)	.214
Blood glucose (mg/dL)	174.50 (131.50, 260.25)	166.50 (130.75, 231.75)	189.50 (134.00, 289.25)	.317
Treatment				
Valve surgery	9 (12.90)	1 (3.60)	8 (19.00)	.126
Bypass surgery	5 (7.10)	0 (0.00)	5 (11.90)	.155
CRRT	22 (31.40)	6 (21.40)	16 (38.10)	.227

*Notes:* Variables statistically significant (*p* < .05) are presented in bold. Continuous variables are expressed as mean ± standard deviation or median (interquartile range) according to normality. Categorical variables are expressed as frequency (percentages). ECMO: extracorporeal membrane oxygenation; APSIII: acute physiology score II: OASIS: oxford acute severity of illness score; LODS: logistic organ dysfunction system; SOFA: sequential organ failure assessment; COPD: chronic obstructive pulmonary disease; CAD: coronary artery disease; MAP: mean arterial pressure; HR: heart rate; SpO2: oxygen saturation; BUN: blood urea nitrogen; WBC: white blood cell count; RBC: red blood cell count; RDW: red cell distribution width; INR: international normalized ratio; PT: prothrombin time; APTT: activated partial thromboplastin time; AST: aspartate aminotransferase; TBIL: total bilirubin; CRRT: continuous renal replacement therapy.

^a^Statistically significant.

The baseline characteristics of patients treated with CRRT were presented in Table 2. The average age of the patients was 61.71 ± 14.98 years, with men accounting for 61.4%. The differences in APSIII, OASIS, LODS, and SOFA between the survival and death patient groups were statistically significant (*p* < .001).

**Table 2.** Baseline clinical data of the patients with sepsis receiving CRRT support.

Variables	Overall (*n* = 513)	Survival (*n* = 229)	Death (*n* = 284)	*p* Value
Age (years)	61.71 (14.98)	60.41 (14.70)	62.76 (15.14)	.077
Sex, male	315 (61.40)	127 (55.50)	188 (66.20)	**.017** ^a^
APSIII	85.63 (25.91)	75.38 (24.00)	93.89 (24.44)	**<.001** ^a^
OASIS	44.00 (35.00,50.00)	38.00 (32.00,47.00)	47.00 (39.00,51.25)	**<.001** ^a^
LODS	9.38 (3.53)	8.22 (3.70)	10.32 (3.10)	**<.001** ^a^
SOFA	12.41 (3.83)	10.70 (3.91)	13.79 (3.16)	**<.001** ^a^
Charlson comorbidity score	6.00 (4.00,8.00)	6.00 (4.00,8.00)	6.00 (4.00,9.00)	.906
Comorbidities, n (%)				
Heart failure	199 (38.80)	83 (36.20)	116 (40.80)	.331
Prior history of hypertension	318 (62.00)	148 (64.60)	170 (59.90)	.310
Atrial fibrillation	64 (12.50)	30 (13.10)	34 (12.00)	.802
Renal insufficiency	250 (48.70)	122 (53.30)	128 (45.10)	.079
COPD	68 (13.30)	23 (10.00)	45 (15.80)	.073
CAD	134 (26.10)	55 (24.00)	79 (27.80)	.383
Stroke	30 (5.80)	11 (4.80)	19 (6.70)	.474
Malignancy	71 (13.80)	31 (13.50)	40 (14.10)	.960
Vital signs				
MAP (mmHg)	73.00 (63.00,88.00)	77.00 (65.00,92.00)	70.00 (60.00,83.25)	**.001** ^a^
HR (bpm)	95.48 (22.05)	92.55 (22.41)	97.84 (21.52)	**.007** ^a^
SpO2 (%)	97.00 (93.00, 100.00)	98.00 (94.00, 100.00)	96.00 (92.00, 100.00)	**.001** ^a^
Laboratory tests				
Lactate (mmol/L)	3.20 (1.80,7.00)	2.40 (1.50,3.60)	4.90 (2.50,9.33)	**<.001** ^a^
Creatinine (mg/dL)	4.07 (2.58)	4.39 (2.99)	3.81 (2.16)	**.012** ^a^
BUN (mg/dL)	46.00 (29.00,72.00)	48.00 (29.00,75.00)	46.00 (29.00,70.25)	.413
WBC (×10^9^/L)	13.20 (7.80,19.20)	11.60 (7.50,18.40)	14.30 (8.57,20.22)	**.032** ^a^
RBC (×10^12^/L)	3.22 (0.80)	3.21 (0.83)	3.22 (0.78)	.847
RDW (%)	16.90 (14.80,18.80)	16.90 (14.90,18.50)	17.00 (14.70,19.20)	.560
Hemoglobin (g/dL)	9.71 (2.35)	9.63 (2.41)	9.77 (2.30)	.505
Platelet (×10^9^/L)	152.00 (80.00, 230.00)	159.00 (95.00, 231.00)	141.00 (73.00, 226.75)	**.030** ^a^
INR	1.70 (1.30,2.50)	1.50 (1.20,2.00)	1.90 (1.40,2.90)	**<.001** ^a^
PT (s)	18.60 (14.50,26.50)	16.90 (13.80,21.60)	20.90 (15.40,31.18)	**<.001** ^a^
APTT (s)	39.50 (32.10,53.90)	36.10 (31.10,46.00)	42.80 (33.38,59.30)	**<.001** ^a^
AST (U/L)	92.00 (43.00, 511.00)	82.00 (30.00, 204.00)	129.50 (71.00, 946.75)	**<.001** ^a^
TBIL (mg/dL)	1.30 (0.60,3.70)	1.30 (0.50,2.80)	1.40 (0.70,4.25)	**.008** ^a^
Blood glucose (mg/dL)	136.00 (100.00, 199.00)	139.00 (106.00, 191.00)	132.00 (92.50, 201.25)	.182
Treatment				
Valve surgery	13 (2.5)	4 (1.7)	9 (3.2)	.461
Bypass surgery	9 (1.8)	3 (1.3)	6 (2.1)	.726

*Notes:* Variables statistically significant (*p* < .05) are presented in bold. Continuous variables are expressed as mean ± standard deviation or median (interquartile range) according to normality. Categorical variables are expressed as frequency (percentages). CRRT: continuous renal replacement therapy; APSIII: acute physiology score III; OASIS: oxford acute severity of illness score; LODS: logistic organ dysfunction system; SOFA: sequential organ failure assessment; COPD: chronic obstructive pulmonary disease; CAD: coronary artery disease; MAP: mean arterial pressure; HR: heart rate; SpO2: oxygen saturation; BUN: blood urea nitrogen; WBC: white blood cell count; RBC: red blood cell count; RDW: red cell distribution width; INR: international normalized ratio; PT: prothrombin time; APTT: activated partial thromboplastin time; AST: aspartate aminotransferase; TBIL: total bilirubin.

^a^Statistically significant.

### Risk factors for mortality in patients with sepsis undergoing ECMO implantation

**Figure 2.**
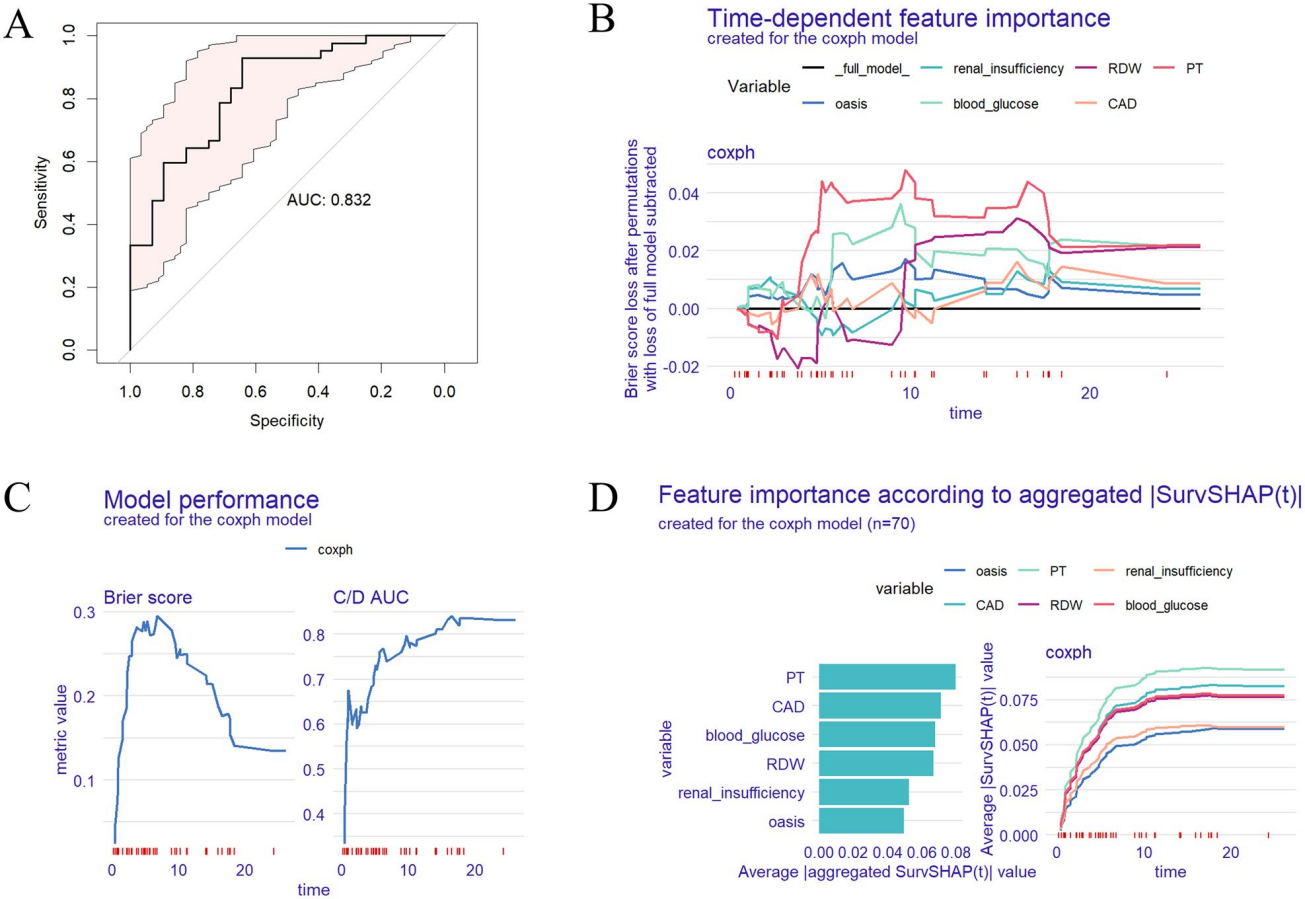
Model-agnostic explanations for the Cox regression model in patients with sepsis undergoing ECMO implantation. (A) ROC curve analysis for predicting death in patients with sepsis undergoing ECMO implantation. (B) Time-dependent feature importance plot created for the Coxph model. (C) Model performance created for the Coxph model. (D) Feature importance according to aggregated |SurvSHAP(t)| created for the Coxph model. ECMO: extracorporeal membrane oxygenation; AUC: area under curve; RDW: red cell distribution width; PT: prothrombin time; CAD: coronary artery disease; C/D AUC: concordance/divergence area under the curve.

A univariate Cox regression analysis was conducted to identify potential risk factors for mortality in patients with sepsis undergoing ECMO implantation. As detailed in Table 3, the results showed that age, OASIS, Charlson comorbidity score, renal insufficiency, COPD, CAD, lactate, creatinine, RBC, RDW, hemoglobin, INR, PT, TBIL, blood glucose and bypass surgery were associated with mortality. (*p* < .1) Subsequently, multivariate Cox regression analysis identified RDW (per 1% increase, HR 1.215, 95% CI 1.048–1.409, *p* = .010), PT (per 1-s increase, HR 1.037, 95% CI 1.007–1.068, *p* = .015), and blood glucose (per 1 mg/dL increment, HR 1.003, 95% CI 1.000–1.005, *p* = .032) as independent prognostic factors. The ROC curve analysis demonstrated robust performance of our Cox regression model (area under the curve [AUC] 0.832; Figure 2(A)). Moreover, the Brier score, a measure of the accuracy of probabilistic predictions, stabilized around 0.1, indicating a good model performance. Furthermore, the Concordance/Divergence Area Under the Curve (C/D AUC) increased from 0.4 to approximately 0.8 over time, reinforcing the model’s consistency and reliability (Figure 2(C)). The time-dependent feature importance plot showed that in the Cox regression model, the importance of different variables on the prediction results varied over time. PT was the most important feature, while other features had lower importance and greater fluctuations (Figure 2(B)). SurvSHAP (t) prediction illustrated significant differences in the feature importance of different variables in the Cox regression model. PT and CAD maintained consistent high importance for model prediction, while variables such as blood glucose and RDW exhibited moderate significance, and renal dysfunction and OASIS had minimal impact (Figure 2(D)).

**Table 3.** Univariate and multivariate Cox regression analysis of risk factors for mortality in sepsis patients undergoing ECMO implantation.

Variables	Univariate		Multivariate	
	HR (95% CI)	*p* Value	HR (95% CI)	*p* Value
Age (per one year increase)	1.019 (0.998–1.040)	.071		
OASIS (per one-point increase)	1.045 (1.007–1.085)	.021	1.029 (0.990–1.069)	.145
Charlson comorbidity score	1.168 (1.025–1.331)	.020		
Renal insufficiency	1.829 (0.932–3.586)	.079	1.852 (0.899–3.813)	.095
COPD	2.963 (1.047–8.386)	.041		
CAD	2.022 (1.099–3.719)	.024	1.831 (0.967–3.469)	.063
Lactate (per 1 mmol/L increase)	1.101 (1.020–1.188)	.013		
Creatinine (per 1 mg/dL increase)	1.099 (0.995–1.213)	.063		
RBC (per 10^12^/L decrease)	0.699 (0.503–0.973)	.034		
**RDW** (per 1% increase)	1.122 (0.990–1.272)	.071	1.215 (1.048–1.409)	**.010** ^a^
Hemoglobin (per 1 g/dL decrease)	0.907 (0.814–1.011)	.079		
INR (per one unit increase)	1.437 (1.099–1.880)	.008		
**PT** (per 1 s increase)	1.034 (1.008–1.060)	.009	1.037 (1.007–1.068)	**.015** ^a^
TBIL (per 1 mg/dL increase)	1.128 (0.993–1.280)	.064		
**Blood glucose** (per 1 mg/dL increase)	1.002 (1.000–1.004)	.048	1.003 (1.000–1.005)	**.032** ^a^
Bypass surgery	3.006 (1.166–7.750)	.023		

*Notes:* Variables statistically significant (*p* < .05) in the multivariate Cox regression analysis are presented in bold. ECMO: extracorporeal membrane oxygenation; HR: hazard ratio; CI: confidence interval; OASIS: oxford acute severity of illness score; COPD: chronic obstructive pulmonary disease; CAD: coronary artery disease; RBC: red blood cell count; RDW: red blood cell distribution width; INR: international normalized ratio; PT: prothrombin time; TBIL: total bilirubin.

^a^ Statistically significant.

### Risk factors for mortality in patients with sepsis treated with CRRT

**Figure 3.**
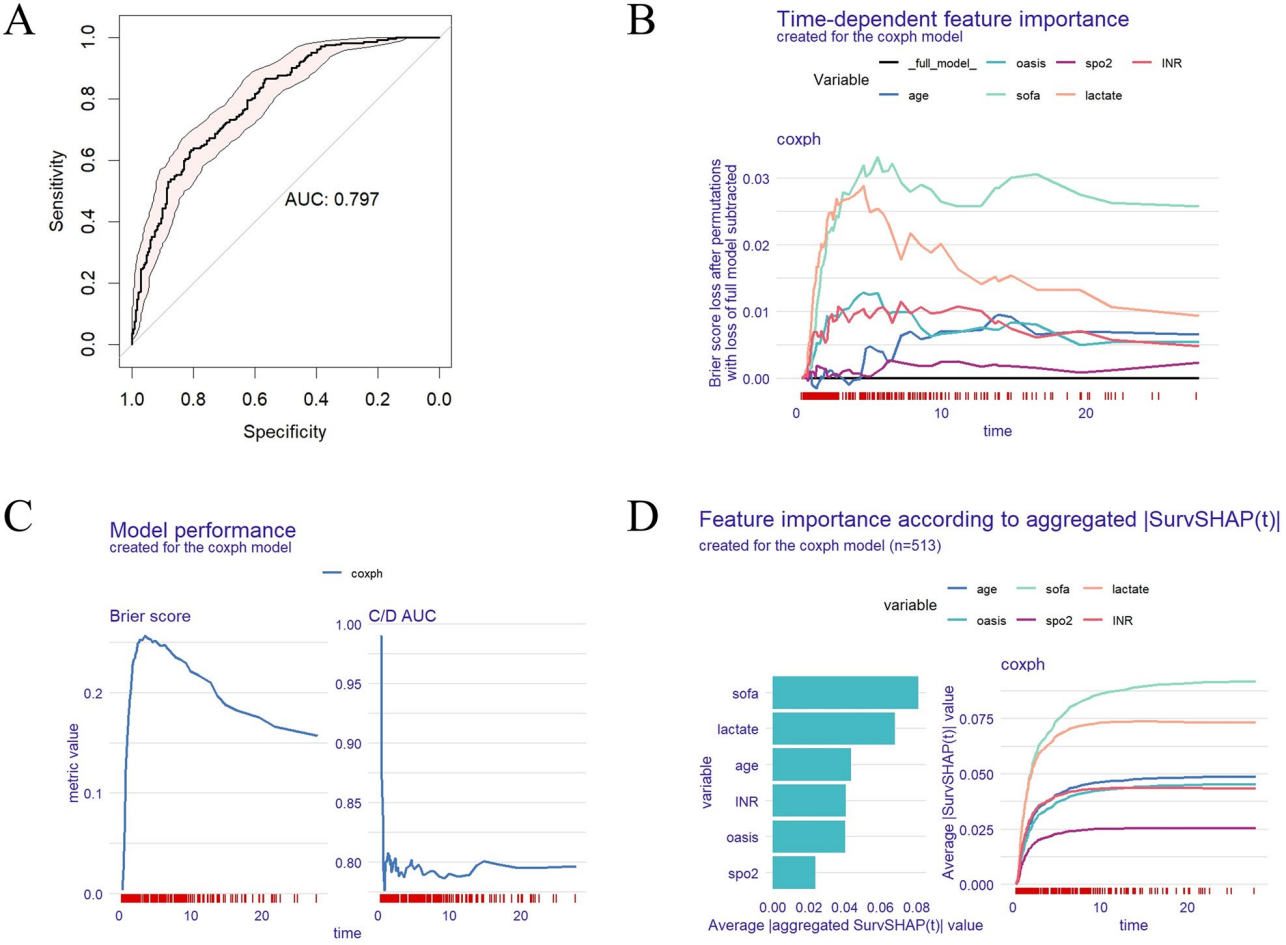
Model-agnostic explanations for the Cox regression model in patients with sepsis undergoing CRRT treatment. (A) ROC curve analysis for predicting death in patients with sepsis undergoing CRRT treatment. (B) Time-dependent feature importance plot created for the Coxph model. (C) Model performance created for the Coxph model. (D) Feature importance according to aggregated |SurvSHAP(t)| created for the Coxph model. CRRT: continuous renal replacement therapy; AUC: area under curve; spo2: oxygen saturation; INR: international normalized ratio; sofa: sequential organ failure assessment; C/D AUC: concordance/divergence area under the curve.

A univariate Cox regression analysis was conducted to identify the potential risk factors for mortality in patients with sepsis treated with CRRT. As shown in Table 4, the analysis revealed associations between mortality and various factors including age, APSIII, OASIS, LODS, SOFA, renal insufficiency, COPD, MAP, HR, SpO2, lactate, creatinine, WBC, platelet, INR, PT, APTT, AST, TBIL, and ECMO. The multivariate Cox regression analysis identified age (per one year increase, HR 1.014, 95% CI 1.005–1.023, *p* = .003), OASIS (per one-point increase, HR 1.019, 95% CI 1.003–1.035, *p* = .019), SOFA (per one-point increase, HR 1.100, 95% CI 1.055–1.147, *p* < .001), SpO_2_ (per 1% decrease, HR 0.980, 95% CI 0.966–0.995, *p* = .009), lactate (per 1 mmol/L increase, HR 1.068, 95% CI 1.045–1.091, *p* < .001), INR (per one unit increase, HR 1.177, 95% CI 1.099–1.260, *p* < .001) as independent risk factors. The ROC curve analysis demonstrated good performance of our Cox regression model with an area under the curve (AUC) of .797 (Figure 3(A)). Model performance was further confirmed by the Brier score, which hovered around 0.15, and the C/D AUC, which remained steady around 0.8 (Figure 3(C)). The plot of time-dependent feature importance revealed SOFA and lactate emerged as the most influential features, whereas other features displayed lower importance and higher variability (Figure 3(B)). Analysis using SurvSHAP (t) prediction revealed SOFA and lactate were identified as the key predictive variables in the Cox regression model, maintaining consistent importance over time. Age and INR showed some relevance but with comparatively lower importance. Conversely, OASIS and SpO2 were identified as the least impactful variables (Figure 3(D)).

**Table 4.** Univariate and multivariate Cox regression analysis of risk factors for mortality in sepsis patients undergoing CRRT.

Variables	Univariate		Multivariate	
	HR (95% CI)	*p* Value	HR (95% CI)	*p* Value
**Age** (per one year increase)	1.007 (0.999–1.015)	.094	1.014 (1.005–1.023)	**.003** ^a^
APSIII (per one-point increase)	1.019 (1.015–1.023)	<.001		
**OASIS** (per one-point increase)	1.051 (1.038–1.064)	<.001	1.019 (1.003–1.035)	**.019** ^a^
LODS (per one-point increase)	1.138 (1.100–1.177)	<.001		
**SOFA** (per one-point increase)	1.164 (1.127–1.201)	<.001	1.100 (1.055–1.147)	**<.001** ^a^
Renal insufficiency	0.727 (0.576–0.919)	.008		
COPD	1.385 (1.007–1.905)	.045		
MAP (per 1 mmHg decrease)	0.992 (0.986–0.998)	.007		
HR (per 1 bpm increase)	1.008 (1.003–1.014)	.002		
**SpO2** (per 1 % decrease)	0.965 (0.951–0.979)	<.001	0.980 (0.966–0.995)	**.009** ^a^
**Lactate** (per 1 mmol/L increase)	1.093 (1.073–1.114)	<.001	1.068 (1.045–1.091)	**<.001** ^a^
Creatinine (per 1 mg/dL decrease)	0.937 (0.893–0.984)	.009		
WBC (per 10^9^/L increase)	1.008 (1.002–1.015)	.011		
Platelet (per 10^9^/L decrease)	0.999 (0.997–1.000)	.013		
**INR** (per one unit increase)	1.238 (1.162–1.319)	<.001	1.177 (1.099–1.260)	**<.001** ^a^
PT (per 1s increase)	1.019 (1.013–1.025)	<.001		
APTT (per 1s increase)	1.007 (1.003–1.010)	<.001		
AST (per 1 U/L increase)	1.000 (1.000–1.0001)	<.001		
TBIL (per 1 mg/dL increase)	1.012 (0.998–1.026)	.084		
ECMO	1.711 (1.017–2.880)	.043		

*Notes:* Variables statistically significant (*p* < .05) in the multivariate Cox regression analysis are presented in bold. CRRT: continuous renal replacement therapy; CI: confidence interval; APSIII: acute physiology score III; LODS: logistic organ dysfunction system; OASIS: oxford acute severity of illness score; SOFA: sequential organ failure assessment; COPD: chronic obstructive pulmonary disease; MAP: mean arterial pressure; HR: heart rate; SpO2: oxygen saturation; WBC: white blood cell count; INR: international normalized ratio; PT: prothrombin time; APTT: activated partial thromboplastin time; AST: aspartate aminotransferase; TBIL: total bilirubin; ECMO: extracorporeal membrane oxygenation.

^a^ Statistically significant.

### Risk factors for mortality in patients with sepsis undergoing combined ECMO and CRRT

**Figure 4.**
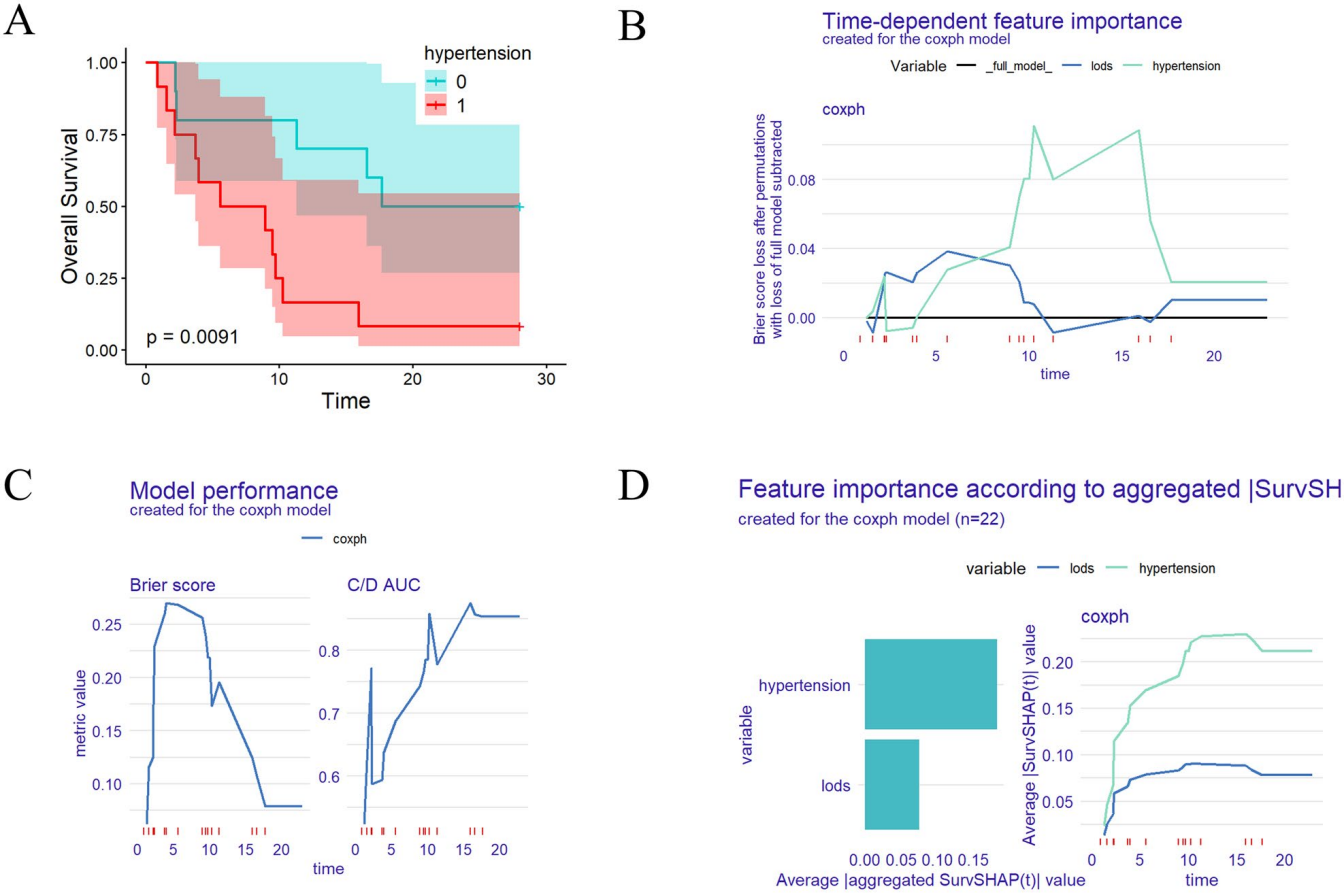
Model-agnostic explanations for the Cox regression model undergoing ECMO implantation and CRRT treatment. (A) ROC curve analysis for predicting death in patients with sepsis undergoing ECMO implantation and CRRT treatment. (B) Time-dependent feature importance plot created for the Coxph model. (C) Model performance created for the Coxph model. (D) Feature importance according to aggregated |SurvSHAP(t)| created for the Coxph model. ECMO: extracorporeal membrane oxygenation; CRRT: continuous renal replacement therapy; AUC: area under curve; LODS: logistic organ dysfunction system; C/D AUC: concordance/divergence area under the curve.

Because patients may be treated with CRRT and ECMO combined, we conducted further analysis to identify independent risk factors for patients with sepsis treated with both CRRT and ECMO. Among the patients included in the final analysis, 22 received ECMO and CRRT (Figure 1). Univariate Cox regression analysis indicated that LODS, prior history of hypertension and CAD were associated with mortality. Subsequently, multivariate Cox regression analysis identified that prior history of hypertension (HR 4.342, 95% CI 1.332–14.153, *p* = .015) remained the independent risk factor for prognosis (Table 5). Consistent with Cox regression analysis, Kaplan–Meier survival analysis further demonstrated a significant association between prior history of hypertension and mortality (Figure 4(A)). Our model’s performance was robust, as evidenced by the Brier score < 0.1 and the C/D AUC > 0.8, indicating accurate probabilistic predictions (Figure 4(C)). The time-dependent feature importance plot illustrated that prior history of hypertension played the most pivotal role influencing predictions, while LODS showed less consistency and greater variability (Figure 4(B)). The SurvSHAP (t) prediction analysis reinforced the central role of prior history of hypertension in the Cox regression model, maintaining stable importance over time, whereas LODS exhibited lower influence (Figure 4(D)).

**Table 5.** Univariate and multivariate Cox regression analysis of risk factors for mortality in sepsis patients undergoing ECMO implantation and CRRT treatment.

Variables	Univariate		Multivariate	
	HR (95% CI)	*p* Value	HR (95% CI)	*p* Value
LODS (per one-point increase)	0.836 (0.688–1.016)	.071	0.855 (0.720–1.016)	.075
**Prior history of hypertension**	4.067 (1.330–12.440)	.014	4.342 (1.332–14.153)	**.015** ^a^
CAD	2.452 (0.880–6.828)	.086		

^a^ Statistically significant.

Variables statistically significant (*p* < .05) in the multivariate Cox regression analysis are presented in bold. HR: hazard ratio; CI: confidence interval; LODS: logistic organ dysfunction system; CAD: coronary artery disease.

## Discussion

In the present study, we found that PT was the key risk factor in patients with sepsis needing ECMO, while the SOFA score was crucial for those requiring CRRT. For septic patients needing both treatments, prior history of hypertension was the primary risk factor.

Sepsis is a pro-inflammatory state causing microvascular changes, pathological vasodilatation and multiorgan failure. Alterations in physiology can occur from sepsis systemic effects, heart, and kidney cross talk, and the impact of care processes [13]. Both ECMO and CRRT play crucial roles in the realm of critical care medicine, serving as life-sustaining, *in vitro* organ support technologies. ECMO is used in sepsis-induced cardiogenic shock, while CRRT provides a consistent filtration of toxic substances, thereby improving renal function. In the treatment of sepsis patients, these two formidable tools often need to collaborate. This is because, while ECMO offers efficient cardiopulmonary support, it possesses an inherent ‘self-damaging’ characteristic that can instigate changes in hemodynamics, provoke hemolysis and stress, and elevate the risk of acute kidney injury. The combination of ECMO and CRRT can effectively augment and maintain hemodynamic stability, mitigate inflammatory storms, rectify internal environmental imbalances, and ultimately facilitate successful critical care rescue [14,15].

Previous studies have demonstrated that factors such as age, blood glucose, CAD, minimum fibrinogen concentration, ECMO duration, cardiopulmonary resuscitation prior to ECMO, CRRT, and initial platelet count were associated with ECMO prognosis [16–18]. In our study, the features were PT, CAD, blood glucose, RDW, renal insufficiency, and OASIS. These findings underscore the importance of variables related to anticoagulation, cardiovascular disease, blood glucose, and renal function. PT reflects the function of the extrinsic coagulation pathway and assesses the levels of prothrombin and related coagulation factors. Prolonged PT may reflect underlying coagulopathy or liver dysfunction, necessitating careful monitoring and potential adjustments in anticoagulant therapy [19,20]. Anticoagulants, such as heparin, are required during VA-ECMO therapy to prevent thrombosis within the equipment; however, anticoagulant therapy also increases the risk of bleeding. For patients with prolonged PT, it is necessary to closely monitor coagulation parameters and adjust the anticoagulant dosage to achieve a balance between preventing thrombosis and reducing bleeding risk. Additionally, patients with sepsis often have multiple organ dysfunction, and liver dysfunction can also lead to prolonged PT. Monitoring liver function during treatment is also significant. However, it is important to acknowledge that ECMO is not a classic indication for sepsis or septic shock management, as its primary indication is in cases of cardiogenic or respiratory failure. As Bréchot et al. demonstrated, ECMO may serve as a rescue therapy specifically in cases of sepsis-induced cardiogenic shock rather than in general sepsis or septic shock [2].

For CRRT, previous research has identified fluid balance, AKI, machine and circuit-related complications, anticoagulation, cardiac comorbidity, time to initiation of CRRT, and liberation patterns as key factors [21–26]. In our study, the features ranked by importance were SOFA, lactate, age, INR, OASIS, and SpO2. These results highlight the significance of focusing on organ failure, anticoagulation, and complications. Studies have indicated that up to 50% of patients with sepsis develop AKI [27–29], leading to poor prognosis and a mortality rate (75%) that is significantly higher than that in patients with sepsis without organ failure [30–32]. It is likely that many sepsis biomarkers may be removed by convection, and therefore, their reliability as markers in patients undergoing CRRT is under question [33]. Interestingly, while INR was independent risk factor for septic patients using CRRT. This also suggested that the rational use of anticoagulants and the management of coagulation functions might also be crucial during treatment.

Due to its simplicity and accuracy, the SOFA score is widely used in intensive care units globally. By assessing the status of six organ systems—respiratory, cardiovascular, central nervous system, renal function, liver function, and coagulation—the SOFA score provides a comprehensive reflection of the overall health status of patients with sepsis. The SOFA score effectively predicts disease progression and mortality risk in patients with sepsis [34]. This study reaffirmed the role of the SOFA score as an independent risk factor in predicting mortality among patients with sepsis undergoing CRRT. Compared to single variable, we consider that the SOFA score provides a more comprehensive reflection of the patient’s condition, thereby offering a more accurate prognostic assessment.

In our study, lactate was found to be independently correlated with higher mortality in patients with sepsis treated with CRRT. Lactic acidosis is associated with high anion-gap metabolic acidosis, resulting in decreased efficacy of vasoactive medications and antibiotics causing potentially prolonged and more severe circulatory failure [35,36]. Many studies show that initial or persistent hyperlactatemia is associated with adverse outcome. In normotensive patients with sepsis, lactate concentration more than 4 mmol/L was found to be independently correlated with higher mortality [37]. And in patients with septic shock, intermediate concentrations of lactate (2–4 mmol/L) or even within the high end of the normal range (1.4–2.3 mmol/L) still indicated poorer prognosis than patients with low normal lactate concentrations [38,39]. Interestingly, our analysis of time-dependent feature importance showed that lactate was more important than SOFA for evaluating risk factors in patients with sepsis treated with CRRT in the early stages. In the middle and late stages, lactate no longer affected the prognosis as the most significant independent risk factor as it did in the early stage.

Additionally, prior studies have shown that age, valvular heart disease, serum albumin levels, and APACHE II and SAPS II scores were associated with the prognosis of ECMO and CRRT treatments [5,40]. Interestingly, in our study, prior history of hypertension emerged as an independent risk factor for patients with sepsis undergoing ECMO and CRRT. This finding is intriguing; on one hand, our small sample size may limit the persuasiveness of this result. On the other hand, it emphasizes the importance of monitoring blood pressure in patients receiving these treatments. Norepinephrine is the recommended first-line vasoactive agent in Cardiorenal syndrome type 5 (CRS-5) patients. However, there is concern for norepinephrine toxicity in patients with CRS-5 due to excessive renal vascular constriction and renal medullary hypoxia [41]. In a sheep sepsis model, vasopressin demonstrates more advantages in enhancing renal function compared to norepinephrine. It neither aggravates renal medullary ischemia and hypoxia nor overly reduces mesenteric blood flow [41]. Owning to a higher mean arterial pressure goal in patients with chronic hypertension, the potential for greater vasoactive medication use and its consequent toxicity should be considered [42]. In 2010, the Kidney Interventions During Membrane Oxygenation study group surveyed 65 participating extracorporeal life support organization centers and found that the most common indication for ECMO-initiated CRRT was fluid overload, accounting for 43% [4]. Patients with hypertension not only tend to have lower baseline cardiovascular function than those with normal blood pressure, making them more prone to cardiac failure and insufficient tissue perfusion. On the other hand, hypertensive patients may tolerate less fluid load, making volume management more challenging.

This study has several limitations. Firstly, it is a retrospective single-center study, which inherently carries limitations such as unrecognized biases, residual confounding risks, and limited generalizability to other centers or countries with different patient populations. Secondly, the timing and methods of ECMO and CRRT implantation were not included in the analysis, which could potentially impact the results. Factors such as the timing of ECMO and CRRT initiation relative to sepsis diagnosis, the background of ECMO and CRRT initiation, the sequence of ECMO and CRRT initiation, and the method of ECMO and CRRT catheter placement may influence the risk of mortality. Thirdly, the smaller number of patients in the ECMO group may affect our interpretation and generalization of the risk factors for this study. Fourth, external verification is lacking. Fifth, Due to data limitations, we are unable to explore the impact of (a) the need for mechanical ventilation, (b) septic shock, and (c) positive blood culture on the results. Sixth, the imbalance in the number of patients among the three groups may have an impact on the results. Although our results are supported by regression analysis, they are not yet ready for direct clinical application but are important exploring these factors.

In summary, we found that RDW, PT, and blood glucose were independent risk factors for patients with sepsis receiving ECMO implantation. For patients with sepsis receiving CRRT treatment, age, OASIS, SOFA, SpO2, lactate, and INR were independent risk factors. Additionally, in patients with sepsis undergoing both CRRT and ECMO treatment, prior history of hypertension was an independent risk factor.

## Supplementary Material

Figure1.tif

Figure2.tif

## Data Availability

The datasets used and analyzed during the current study are available from MIMIC-IV.
