# Supplementary figures and images for: Risk factors for mortality in patients with sepsis on extracorporeal membrane oxygenation and/or continuous renal replacement therapy: a retrospective cohort study based on MIMIC-IV database
Source: Ren Fail. 2024 Dec 4;46(2):2436106. doi: 10.1080/0886022X.2024.2436106 (PMC11619025; doi:10.1080/0886022X.2024.2436106)

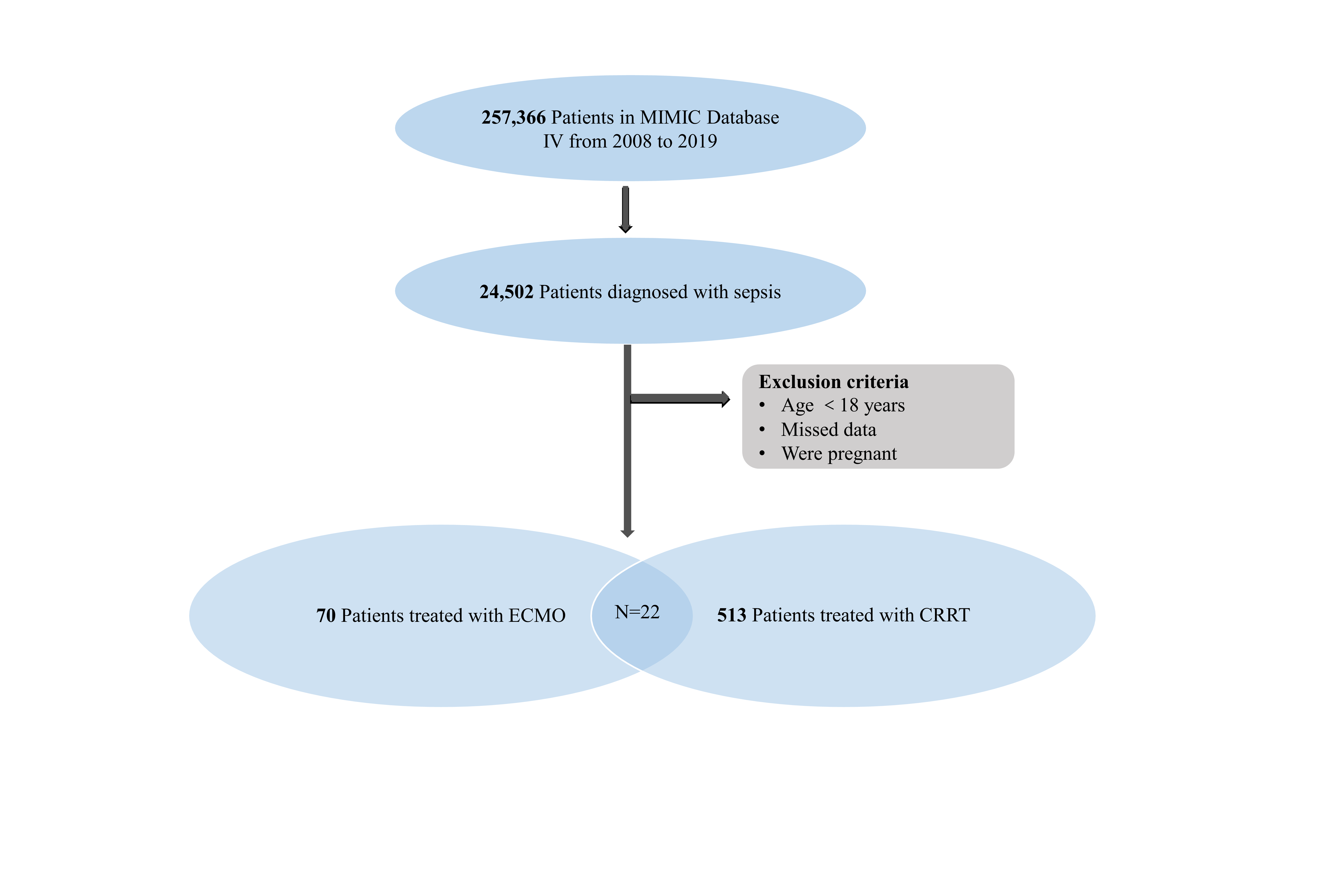

Supplement: Figure1.tif [file IRNF_A_2436106_SM5809.tif]

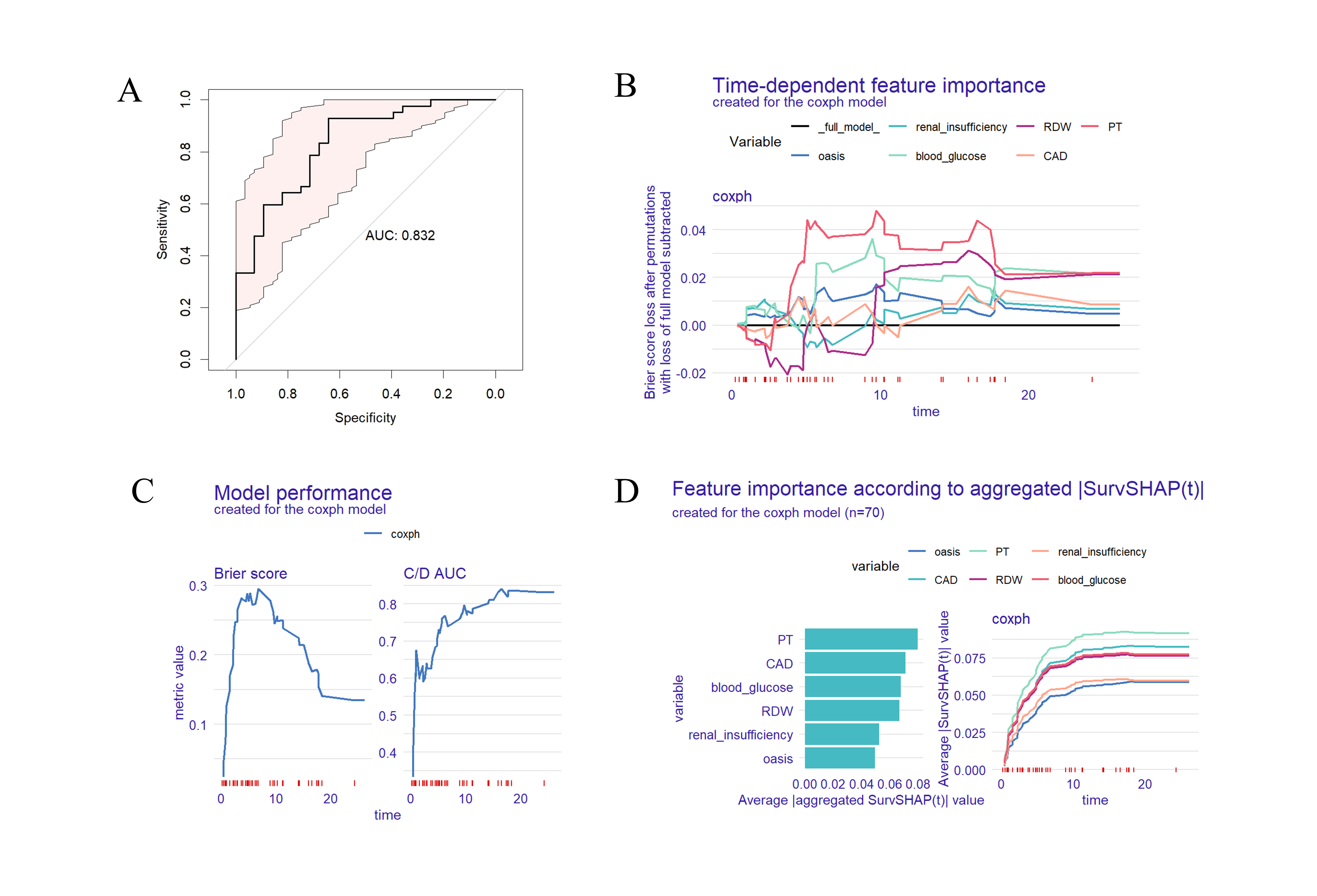

Supplement: Figure2.tif [file IRNF_A_2436106_SM5808.tif]
